# Addressing Contaminants of Emerging Concern in Aquaculture: A Vacuum Membrane Distillation Approach

**DOI:** 10.3390/membranes15050127

**Published:** 2025-04-24

**Authors:** Claudio Marcos Eugênio Malaghini, Jussara Garcez, Rodrigo Hoff, Alan Ambrosi, Katia Rezzadori

**Affiliations:** 1 Department of Food Science and Technology, Federal University of Santa Catarina, Av. Ademar Gonzaga, 1346, Itacorubi, Florianópolis 88034-000, SC, Brazil; cutighini@gmail.com; 2 Department of Chemical and Food Engineering, Federal University of Santa Catarina, Florianópolis 88040-900, SC, Brazil; jussaragarcezz@gmail.com (J.G.); alan.ambrosi@ufsc.br (A.A.); 3 Federal Laboratory of Agricultural Defense—LFDA/RS, Ministry of Agriculture, Livestock and Food Supply of Brazil, Rua João Grumiche 117, São José 88102-600, SC, Brazil; rodrigo.hoff@agro.gov.br

**Keywords:** emerging pollutants, wastewater treatment, membrane distillation

## Abstract

The presence of contaminants of emerging concern (CECs) in agricultural and fisheries water has raised significant environmental and health concerns. Vacuum membrane distillation (VMD) has shown promise as an effective method for removing non-volatile contaminants, such as CECs, from water. This study presents a novel application of a bench-scale VMD unit to treat water from Lagoa da Conceição, Florianópolis, Brazil, using microporous membranes (0.22 µm) under the following optimized conditions: 75 °C, a flow rate of 24 L·h^−1^, and a vacuum pressure of −640 mmHg. The system demonstrated remarkable performance in removing several key antimicrobials, including sulfamethoxazole, ciprofloxacin, azithromycin, and clindamycin (500 μg·L^−1^), with rejection rates of 99.1%, 98%, 99.9%, and 99%, respectively, and an average flux of 7.08 L·m^−2^·h^−1^. Additionally, the VMD unit achieved a substantial 99.98% salt rejection. Ecotoxicity tests revealed low toxicity for sulfamethoxazole, ciprofloxacin, and azithromycin but high toxicity for clindamycin, while human risk assessment indicated moderate-to-high risks for ciprofloxacin and clindamycin. The findings highlight the potential of VMD as an effective and sustainable technology for the removal of CECs and biocompounds, enhancing water safety and reducing environmental hazards. This study offers a promising solution for addressing water contamination on a broader scale.

## 1. Introduction

Contaminants of emerging concern (CECs) are substances that have recently been recognized as potential environmental and health hazards, often due to advances in detection technology [1,2]. Emerging contaminants, such as sulfonamides (including sulfamethoxazole, sulfaguanidine, sulfanilamide, sulfadimethoxine, sulfathiazole, sulfamethazine, sulfaquinoxaline, sulfadiazine, sulfamerazine, and trimethoprim), are well-recognized and commonly used in human and animal health as antimicrobials.

In addition, other substances, such as pharmaceutical and personal care products, nanomaterials, hormones, endocrine disruptors, perfluorinated chemicals, microplastics, nanoplastics, and micropollutants (which, though present in low concentrations, remain potentially harmful), can contribute to the development of microbial resistomes. Furthermore, CECs typically originate from products commonly consumed by the public or mass-produced by industries [1,2].

Although they are not officially regulated by environmental policies or legislation, they have been detected in water bodies, sediments, and living organisms [1,2]. Both conventional and emerging contaminants have multifactorial origins and transversal implications for human health. Nowadays, One Health is a global approach and recognizes that the health of humans, domestic and wild animals, plants, and the environment (including ecosystems) are closely interconnected and interdependent [3]. In this context, studies investigating complex and highly productive ecosystems, such as the Lagoa da Conceição in Florianópolis, Santa Catarina state, Brazil (27°37′01.7″ S latitudinally and 48°27′10.0″ W longitudinally), are important and necessary. Fishing in the Lagoa da Conceição has great socio-economic relevance for Santa Catarina Island, where the artisanal fishing community has been established since the early days of colonization. The capture of mullet, shrimp, and crabs is crucial both for artisanal fishing and for the diet of local fishers [4].

This area, a popular tourist destination and a vital source of livelihood, has faced significant challenges from water leaching and pollution in recent years. In 2021, heavy rainfall caused the evapoinfiltration lagoon at the wastewater treatment plant (WWTP) managed by Companhia Catarinense de Águas e Saneamento (CASAN) to rupture, leading to an environmental disaster that severely impacted the Lagoa da Conceição area.

Studies by Da Silva et al. [5] in strategic points of the Lagoa da Conceição, after the rupture of the evaporation pond in 2021, initially identified 35 ECs, along with detected and quantified compounds (such as caffeine, ciprofloxacin, clindamycin, diclofenac, sertraline, and sulfamethoxazole). Their presence was observed across various ecological environments, including water, sediments, and aquatic organisms. The high frequency of detection of various ECs suggests a significant risk to the estuarine biota due to the potentially toxic effects described in the literature [5]. Socio-demographic and environmental changes increase the population’s vulnerability to EC exposure, emphasizing the relevance of a One Health approach to address public health challenges.

In this context, new strategies and technologies to mitigate associated risks and ensure the preservation and sustainability of aquatic ecosystems are demanded [5]. Among the promising solutions, membrane separation processes have emerged as effective methods for water decontamination, demonstrating efficiencies exceeding 99.5% in saline waters [6]. Specifically, vacuum membrane distillation (VMD) offers advantages by leveraging differences in the physical properties of materials, such as the liquid and vapor states of water, to achieve separation [7]. Unlike conventional thermal desalination technologies (e.g., multi-stage flash and multi-effect distillation), VMD operates at relatively low temperatures (30–80 °C), which significantly reduces energy consumption [7]. In comparison to reverse osmosis (RO), another widely used membrane-based technique, which has a removal efficiency close to that of VMD, the implementation cost of both technologies can vary significantly depending on the specific application and operational conditions. RO, a high-pressure membrane-based technology, generally has a lower unit cost for water purification, with reports indicating costs as low as USD 0.28/m^3^. However, the energy consumption for RO is relatively high due to the pressure required for the process, especially when treating water with high salinity. In contrast, MD operates at lower pressures and relies on thermal gradients for separation, resulting in lower energy consumption when heat is provided by renewable or industrial waste sources. Furthermore, MD could offer significant advantages in terms of water recovery and zero-liquid discharge scenarios, which are not always achievable with RO. Moreover, while RO is affected by high feed concentrations, MD’s efficiency is influenced by changes in feed concentration, offering potential flexibility in certain applications [7,8,9].

Recent research has demonstrated the potential of VMD for removing CECs and decontaminating water. For instance, Zhang et al. [10] assessed the efficiency of VMD in treating wastewater from traditional Chinese medicine processing, achieving reductions exceeding 97% for chemical oxygen demand, total nitrogen, total phosphorus, and ammonia. Studies on the performance of VMD with PTFE membranes, as reported by Woldemariam [7], have shown promising results, with a high salt rejection rate of 99.74% in saline solutions. In terms of antimicrobial removal, VMD has exhibited variable rejection efficiencies, with average rejection rates ranging from 92% to 99% for sulfamethoxazole and 37% to 98% for ciprofloxacin. Furthermore, to enhance the removal of CECs, an approach integrating the Ag-PTFE membrane into the MD system significantly accelerated the degradation of ibuprofen, achieving 81% removal rate within 2 h, highlighting its efficiency in removing emerging contaminants [11].

Despite these promising results, the research progress of traditional membrane separation methods and membrane distillation technology in the context of CEC removal remains fragmented. This study addresses this gap by presenting a comprehensive evaluation of VMD’s efficiency in removing a range of CECs, thus contributing to a better understanding of its potential compared to conventional methods and demonstrating its relevance for large-scale water treatment applications.

Therefore, this study aims to evaluate the performance of the VMD process for the removal of CECs (specifically antimicrobial) from aquaculture water, based on the physicochemical properties of the water from Lagoa da Conceição, located in Florianópolis, SC, Brazil, and analyze the risks for the human health. Additionally, the study sought to investigate the ecotoxicity of these compounds on lettuce (*Lactuca sativa* L.) and assess the potential human health risks associated with exposure to these contaminants in water.

## 2. Materials and Methods

### 2.1. Materials, Reagents, Standards, and Solutions

Analytical standards (purity ≥ 98%) of sulfamethoxazole, ciprofloxacin, azithromycin, and clindamycin hydrochloride were provided by Fluka (Darmstadt, HE, Germany).

All the solvents used were of chromatographic grade, and the reagents were of analytical grade. Acetone, acetonitrile (ACN), and methanol (MeOH) were supplied by JT Baker Chemical Co. (Phillipsburg, PA, USA). Acetic acid (AA) and formic acid (FA) were supplied by Tedia Co. (Fairfield, OH, USA), and ammonium acetate was purchased from Sigma Aldrich Co. (St. Louis, MO, USA). Sodium chloride was obtained from Êxodo Científica (São Paulo, SP, Brazil). Ultrapure water (type I) (minimum resistivity 18.2 MΩ cm) was obtained from a Millipore Milli-Q ^®^ purification system (Molsheim, GES, France).

Saline sodium chloride solution was used (Crystal Sodium Chloride 1 Kg P.A.ACS Êxodo Científica, Brazil) at a concentration of 27 g·L^−1^, and solutions containing the micropollutants were prepared at a concentration of 500 μg·L^−1^. The solutions were prepared in demineralized water using a reverse osmosis system.

The membrane used for the permeation tests was a hydrophobic PTFE (polytetrafluoroethylene) membrane with an average pore diameter of 0.22 µm, purchased from Merck Millipore Ltd. (Darmstadt, HE, Germany).

For solid phase extraction (SPE), Strata-X ^®^ SPE cartridges (6 mL, 200 mg, 30 µm) were obtained from Phenomenex (Torrance, CA, USA).

### 2.2. Sample Preparation for Permeation Tests

Preliminary water desalination tests were carried out to understand how the membrane unit and the vacuum membrane distillation process work. These studies were performed using a saline solution (27 g·L^−1^ NaCl), based on the average values found in the literature for brackish water, to simulate the salinity of the water in Lagoa da Conceição [12,13] and the Lagoa da Conceição water collected according to previous study performed by our group [5].

For the subsequent micropollutant removal tests, saline solutions with antimicrobials were prepared with Milli-Q ultrapure water for each of the analytes (sulfamethoxazole, ciprofloxacin, azithromycin, and clindamycin) at a concentration of 500 μg·L^−1^.

### 2.3. Vacuum Membrane Distillation Process

#### 2.3.1. Equipment, Membrane Specification, and General Description

**Figure 1:**
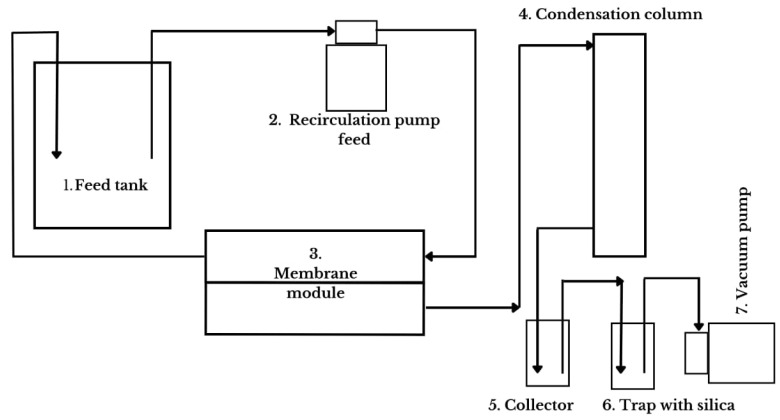
Schematic of the vacuum membrane distillation bench system.

A bench-top flat-sheet VMD permeation system with a membrane area of 0.0086 m^2^ operates based on the principle that a certain volume of sample solution was placed in the feed tank, which was heated to the required temperature using a heating source. The solution was pumped into the VMD module. Meanwhile, the tube section of the VMD module was maintained under negative pressure, created by a vacuum system. As a result, water molecules in the solution migrated across the boundary layer to the membrane surface, where they evaporated into water vapor. The vapor passed through the membrane pores and reached the permeate side, driven by the vapor pressure difference caused by the transmembrane temperature difference. The vapor was condensed by cooling water in the condenser and collected in the collector. The remaining solution continued to concentrate in the system and completed the distillation process. Its configuration included a feed chamber, a hydrophobic membrane, and a permeate chamber connected to a vacuum pump [6], according to the diagram shown in Figure 1.

Where:

1. Feed Tank: double-jacketed borosilicate glass feed tank; it is heated, and samples are added to initiate the process, serving as the initial feed point;
2. Recirculation pump feed (SFDPA2-015-060, Seaflo, Xiamen, China): This pump is responsible for recirculating the solution between the feed and the membrane module. The pump uses a Variac transformer to control the flow rate of the recirculation system, which can be visualized using a rotameter that measures the flow rate;
3. Membrane module: A support structure that holds the membrane through which the solution recirculates at the selected temperature. Above the membrane, a hot aqueous solution flows, and what permeates the membrane—a solution/solvent in a vapor state—flows to the Serpentine Graham condenser;
4. Condensation column (Serpentine Graham): using a 40% ethylene glycol solution at 0 °C, the condenser is responsible for precipitating the solution/solvent so it can be collected in the collector;
5. Collector: where the permeate or distilled solution is collected;
6. Trap with silica: a vacuum pump buffer container containing silica to retain vapor;
7. Vacuum pump (Q-355B, Quimis, São Paulo, Brazil): responsible for creating negative pressure in the system (−640 mmHg).

The membrane used in the tests was the hydrophobic flat-sheet Fluoropore membrane (Merck Millipore), made of polytetrafluoroethylene (PTFE), with the characteristics shown in Table 1. The membranes were pre-cut to fit the module and used without any pre-treatment.

#### 2.3.2. Determining VMD Membrane Performance

Initially, preliminary tests were conducted with a saline solution of NaCl 27 g·L^−1^, as defined in item 2.2 to study the equipment’s flow rate as a function of the feed temperature and vacuum pressure. Based on the literature [14], feed temperatures of 65 °C and 75 °C were studied, and the permeate was condensed using a circulating bath of 40% ethylene glycol at a temperature of 0 °C. The vacuum pump pressure was −640 mmHg, which was the maximum achieved by the pump used in the experiment.

Subsequently, permeation assays were performed for the removal of antimicrobials (sulfamethoxazole, ciprofloxacin, azithromycin, and clindamycin) at a concentration of 500 μg·L^−1^. The antimicrobials were evaluated individually and concurrently in triplicate assays. These antimicrobials were chosen once they were found at different points in Lagoa da Conceição and at higher concentrations, according to studies conducted by Da Silva et al. [5]. The concentration of 500 μg·L^−1^ was overestimated to allow quantification by chromatography. The operational conditions used were the same as those in the preliminary tests, with the feed temperature set at 75 °C, vacuum pump pressure at −640 mmHg, and feed flow rate fixed at 24 L·h^−1^. All tests were performed by fixing the total permeation time at 2 h, with feed recirculation. During each experiment, permeate samples were collected every 15 min over 120 min, i.e., eight reference points were collected at 15, 30, 45, 60, 75, 90, 105, and 120 min. All permeation experiments were performed in triplicate.

The permeate flux, J (L·h^−1^·m^−2^) of each experiment, was determined using Equation (1)

(1) J=V/t · A 

where:

*J* is the permeate flux (L·h^−1^·m^−2^);
*V* is the collected permeate volume (L);
*t* is the sample collection time (h);
*A* is the area in m^2^.

In the preliminary tests, VMD performance was expressed by the rejection rate (%) using a saline solution (NaCl 27 g·L^−1^) as the feed, with measurements carried out by electrical conductivity (CG2000, Gehaka, São Paulo, Brazil) and compared to a salinity curve. Antimicrobial analysis was carried out. Samples were collected from the feed solution before the start of the VMD process, from the permeate at the end of the process, and from the concentrate, which consisted of the portion that did not pass through the membrane and remained on the feed side, as shown in Equation (2):

(2) $$EF\left(\%\right)=\frac{Cinitial-Cfinal}{Cinitial}\;\cdot \;100$$

where:

*Cinitial* is the initial concentration of the analyte or particles in the feed solution;
*Cfinal* is the final concentration of the analyte or particles in the solution (permeate).

### 2.4. Antimicrobial Quantification

#### 2.4.1. Sample Preparation by Solid Phase Extraction (SPE)

Solid phase extraction (SPE) was used to concentrate solution samples. Strata-X^®^ SPE cartridges were first conditioned with 3 mL of MeOH containing 50 mM·L^−1^ of AA, 3 mL of acetone containing 50 mM·L^−1^ of AA, and 3 mL of water containing 5% MeOH, at a flow rate of approximately 5 mL·min^−1^.

After conditioning, the sample (500 mL) was applied to the SPE cartridge. Once the sample had fully eluted, the cartridge was dried for 5 min under vacuum. The analytes were eluted with 3 mL of MeOH containing 50 mM of AA, followed by 3 mL of acetone containing 50 mM of AA. The collected extract was evaporated to dryness using a water bath at 40 °C under a gentle nitrogen flow.

The extract was reconstituted with H_2_O:ACN (95:5) to a final volume of 1 mL. After centrifugation, an aliquot of the supernatant (200 µL) was transferred to a borosilicate glass vial with an insert and injected into an LC-MS/MS system [15].

#### 2.4.2. Analysis by LC-MS/MS

An HPLC 290 Infinity system (Agilent Technologies, Waldbronn, Germany) coupled to a QTRAP^®^ 5500 hybrid triple quadrupole linear ion trap mass spectrometer (Sciex LLC, Framingham, MA, USA) was used. The system featured an electrospray ionization source operating in both positive (ESI+) and negative (ESI-) modes. Analyst 1.6.2 and MultiQuant software (Sciex, Foster City, CA, USA) were employed for data acquisition and processing, respectively.

A chromatographic column C18 LUNA (50 mm × 2.0 mm; 3.0 μm particles, 100 Å pore size) was used, preceded by a C18 guard column (4.0 mm × 3.0 mm) from Phenomenex (Torrance, CA, USA). The mobile phase A consisted of ultrapure water, while mobile phase B was methanol (MeOH), both containing 0.1% acetic acid and 5 mmol L^−1^ ammonium acetate as additives. The elution gradient was programmed as follows: 95% A (1–8 min), 10% A (8–12 min), and 95% A (13–17 min), with an additional 2 min equilibration period between injections. The injection volume was fixed at 10 µL, and the flow rate was 0.3 mL·min^−1^, with the column maintained at 40 °C. Chromatographic conditions were established, as described by Jank et al. and Hoff et al. [15,16].

The ionization parameters were set as follows: ion spray voltage (IS) at 4500 V; curtain gas at 20 psi; nebulizer gas (GS1) and auxiliary gas (GS2) both at 45 psi; and source temperature maintained at 500 °C. Nitrogen was used for nebulization and collision. Specific mass spectrometry parameters for each analyte were optimized in previous studies [16] and confirmed for this method. Data acquisition was performed in selected reaction monitoring (SRM) mode, ensuring sufficient points (≥2) for quantification and confirmation of each analyte.

### 2.5. Plant Ecotoxicity Test with Lactuca sativa L.

To evaluate the toxic effects of antimicrobial solutions and water obtained after membrane permeation (VMD), phytotoxicity assays were conducted using lettuce seeds (*Lactuca sativa* L.) as the test organism. The experiments followed standardized protocols and methodologies recommended by the OECD (2003) and US EPA (1996), with adaptations by Sobrero and Ronco [17].

The assays were conducted in Petri dishes (90 mm in diameter) with filter paper (pore size 14 µm) as a substrate. Twenty non-pelleted lettuce seeds were placed in each dish and moistened with 4 mL of each sample. The dishes were sealed with transparent film to maintain humidity, watered daily with 2 mL of each sample, and kept in an environment at 20 ± 1 °C with a 12:12 h light: dark photoperiod for a 120 h exposure period. Germination was monitored every 24 h, and effective root emergence was considered the germination criterion.

At the end of 120 h, the radicle and hypocotyl lengths of *L. sativa* were measured using a digital caliper. Tests were performed in duplicate, following literature protocols [17] and toxicity calculations according to Marcu et al. and De Andrade et al. [18,19].

The plate arrangement was as follows:

1. Control A and control B (distilled water);
2. Sulfamethoxazole A and sulfamethoxazole B;
3. Ciprofloxacin A and ciprofloxacin B;
4. Azithromycin A and azithromycin B;
5. Clindamycin A and clindamycin B;
6. Mixture A and mixture B.

In addition to germination percentage, the following indices were evaluated: germination index (Equation (2)), normalized residual germination percentage index (Equation (3)), and normalized residual root elongation percentage index (Equation (4)). These indices serve as indicators of toxicity levels for *Lactuca sativa* L. and other seeds, based on De Andrade et al. [19]. The germination index, normalized residual germination percentage index (*NGI*), and normalized residual radical elongation percentage index (*NGR*) were calculated using Equations (3)–(5) below.

(3) $$GI\;(\%)=\frac{RSG(\%)\;\cdot \;RGR(\%)}{100}$$

where:

*RSG*: Relative seed germination

(3a) $$RSG(\%)=\frac{No.\;of\;seeds\;germinated\;with\;sample}{No.\;of\;germinated\;seeds\;in\;the\;negative\;control}\;\cdot \;100$$

*RGR*: Relative growth of the radicle

(3b) $$RGR(\%)=\frac{Average\;radicle\;length\;with\;sample}{Average\;radicle\;length\;in\;the\;control}\;\cdot \;100$$

*NGI*: Normalized Residual Germination Percentage Index

(4) $$NGI\%=\frac{Germy-Germcontrol}{Germcontrol}$$

where:

*Germy* is the average percentage of germinated seeds in each sample;
*Germcontrol* is the percentage of germinated seeds in the control.
*NGR*: Normalized Residual Radical Elongation Percentage Index

(5) $$NGR(\%)=\frac{alongy-alongcontrol}{alongcontrol}$$

where:

*alongy* is the average radicle length of the germinated seeds in each sample;
*alongcontrol* is the average radicle length of the germinated seeds in the control.

According to De Andrade et al. [19], the NGI and NGR indicate the level of toxicity and are classified into the following categories:

Greater than 0: hormesis (beneficial dose for the test organism);

- 0 to −0.25: low toxicity;
−0.25 to −0.5: moderate toxicity;
−0.5 to −0.75: high toxicity;
−0.75 to −1.0: very high toxicity.

For each dataset obtained, normality and variance homogeneity tests were performed (Kolmogorov–Smirnov and Levene’s, respectively). Differences between treated and untreated groups were evaluated using ANOVA followed by Dunnett’s post-test (α = 0.05) for normal data and Kruskal–Wallis followed by Dunn’s post-test for non-parametric data (α = 0.05), comparing the treated groups to the respective negative controls (Sigmaplot 11.0, San Diego, CA, USA).

### 2.6. CECS Human Risk Analysis

The acute risk was assessed based on the calculation of the estimated daily intake (*EDI*) of CECs for water [19], followed by a comparison with their respective acute reference doses (*RfD*) provided by Brazilian regulations. CECs concentration was obtained from Table S1. The estimated daily intake (*EDI*) was obtained using Equation (6):

(6) $$EDI=IR\;\cdot \;\frac{CC}{BW}$$

where *IR* is the water consumption rate by consumers (kg·day^−1^), *CC* is the concentration of CECs (ng·kg^−1^), and *BW* is the average body weight of consumers (kg). For *CC*, the minimum and maximum detected concentrations were considered.

In addition, the estimated average daily dose (*ADD*) in ng·kg^−1^/body weight^−1^·day^−1^ and the hazard index (*HI*) of quantifiable CECs for children (2 years), adult men (30–34 years), and adult women (30–34 years) were calculated using the method described by De Andrade et al. [19], based on Equation (7), estimated average daily dose (*ADD*), and Equation (8), the hazard index (*HI*), respectively:

(7) $$ADD=\frac{MC\;\cdot \;AFC\;\cdot \;EF\;\cdot \;ED}{AT\;\cdot \;BW}$$

(8) $$HI=\frac{ADD}{RfD}$$

where *MC* is the maximum concentration of CECs in the water sample (ng·kg^−1^), *AFC* is the amount (kg) of water consumed per capita per day (35 mL per kg per day, which corresponds to an average of 2.2 kg·day^−1^ [20]), *EF* is the exposure frequency (365 days), *ED* is the exposure duration (12 years for children and 70 years for adults), *BW* is the average body weight of the subject (kg), *AT* is the average time (*EF* x *ED*), and *RfD* is the reference dose for the individual compound (ng·kg^−1^/day) [19]. The data on the average annual per capita water consumption in Brazilian households was considered to be 2.2 kg·day^−1^, as per data obtained by Lucchesi et al. [20]. The values were considered for a 35-year-old individual (median life expectancy in Brazil), with a weight of 70 kg (the Brazilian average is around 68 kg), and daily exposure (daily water consumption).

## 3. Results and Discussion

### 3.1. Permeation Tests

#### 3.1.1. Preliminary Tests for Temperature Process Definition

**Figure 2:**
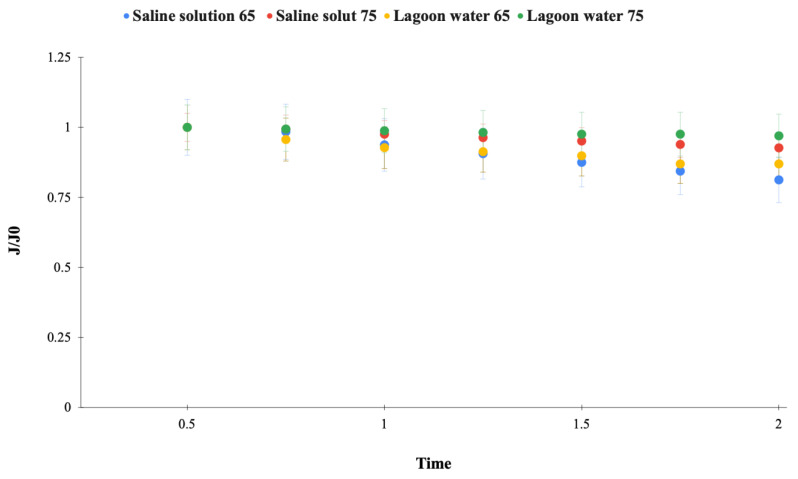
Normalized solution flow in PTFE membrane. Test conditions: temperature 65 and 75 °C, vacuum pressure −640 mmHg, feed permeate flux rate 24 L·h^−1^, NaCl concentration approximately to 27 L·h^−1^, (⧳) saline solution at 65 °C, (⧳) saline solution at 75 °C, (⧳) lagoon water at 65 °C, (⧳) lagoon water at 65 °C, vacuum pressure of −640 mmHg, and feed flow rate of 24 L·h^−1^.

Initially, tests were carried out with a saline solution (27 g·L^−1^), Lagoa da Conceição water (properly), temperatures of 65 and 75 °C, a vacuum pressure of −640 mmHg, and a feed flow rate of 24 L·h^−1^. Permeation and rejection salt results are presented in Figure 2 and Table 2.

Figure 2 shows the variation in permeate flux over time within two hours of the process. During the operation of VMD, the performance was stable, with the salt rejection exceeding 99%. Higher flux was observed at 75 °C for both types of feed solutions, owing to the increased driving force of the process. This increase in permeate flow occurs, because the increase in feed temperature leads to greater mass transfer through the membrane, i.e., greater vapor passage. As the temperature of the feed water rises, it increases the temperature difference between the sides of the membrane, which increases the driving force of the process for the vaporization of water on the pore surface and its consequent permeation through the membrane. Studies carried out by Alawad et al. [21] showed that the feed temperature has a substantial impact on the system’s productivity, showing a notable improvement (415% gain when increasing from 50 to 90 °C). On the other hand, at 75 °C, there was a reduction in permeate flux of around 25% in the first 30 min of the process. This trend is expected in membrane processes, as at the start of the process, a concentration polarization layer forms. In this layer, solutes from the feed concentrate on the membrane surface, reducing the local vapor pressure of the liquid in equilibrium with the feed. This also impacts fluid dynamics and heat transfer [22]. Additionally, a temperature polarization layer forms, reducing the temperature gradient between the two sides of the membrane and leading to a decrease in permeate flux [23]. After 2 h of processing, for both temperatures, a further drop in flux was observed, compared to the initial one, reaching a higher reduction in higher temperatures. It is possible that, as the feed is recirculated during the process, a slight increase (2.2%) in the salt concentration on the feed side occurs, which further hinders the vapor’s ability to permeate through the membrane pores.

Despite the differences in salinity, no significant variation was observed between the feed solutions tested (saline solution and Lagoa da Conceição water). Therefore, a saline solution was used in the subsequent experiments to eliminate the presence of other compounds that could interfere with the rejection results. Additionally, a temperature of 75 °C was selected for the final experiments.

Table 2 shows the analytical results of the feed samples and permeate collected after the VMD experiments. The conductivity and salt concentration values show that salt rejection was around 99% in both the temperatures and solution feed.

Despite the differences in salinity, no significant variation was observed between the feed solutions tested. Therefore, a saline solution was used in the subsequent experiments to avoid the presence of other compounds that could interfere with the rejection results.

#### 3.1.2. Individual and Mixture Antimicrobials Tests

The rejection tests for the antimicrobials sulfamethoxazole, ciprofloxacin, azithromycin, and clindamycin, individually filtered, were performed at 75 °C, with a flow rate of 24 L·h^−1^ and a vacuum pressure of −640 mmHg, at a concentration of 500 μg·L^−1^.

**Figure 3:**
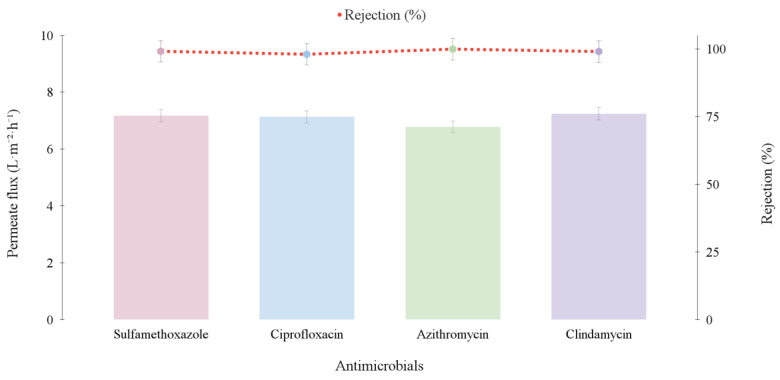
Permeate flux and rejection rate of each antimicrobial evaluated individually.

Initially, the solutions containing the antimicrobials were evaluated individually, with one permeation experiment conducted for each antimicrobial in triplicate. Subsequently, a solution containing all studied antimicrobials together was tested. Figure 3 presents the data on average permeate flux (L·m^−2^·h^−1^) and rejection (%) for each antimicrobial individually.

The test conditions were micropollutant concentration equal to 500 μg·L^−1^, temperature of 75 °C, vacuum pressure of −640 mmHg, and feed flow rate of 24 L·h^−1^.

The average permeate flux obtained after 2 h of permeation for the antimicrobials tested sulfamethoxazole, ciprofloxacin, azithromycin, and clindamycin was 7.17 L·m^−2^·h^−1^, 7.13 L·m^−2^·h^−1^, 6.78 L·m^−2^·h^−1^, and 7.24 L·m^−2^·h^−1^, respectively. These values showed no statistical difference (*p* < 0.05) among them.

Regarding the rejection of antimicrobials, it was observed that the membrane presented a high rejection for all analytes, with a retention rate exceeding 99% (Figure 3). The lowest rejection rate was observed for ciprofloxacin (98%), which could be related to its molar mass, one of the lowest (331.35 g·mol^−1^). The primary mechanism for CEC removal in membrane distillation (MD) is the volatility of the compounds. MD’s performance is significantly influenced by volatility, because mass transfer occurs in the vapor phase under normal operating conditions without pore wetting. Therefore, the higher the volatility of a compound, the more likely it is to permeate the membrane. Volatility is often quantified by the compound’s pKH, which is derived from Henry’s law constant (H) [24]. Costa et al. [25] conducted a review on the performance of MD in the removal of various trace organic compounds. The authors reported the highest removal efficiencies (99–100%) for compounds with a pKH greater than 12. This indicates that, as pKH increases (reflecting lower volatility), the impact of CECs’ hydrophobicity on removal efficiency decreases. In contrast, compounds with lower pKH values showed a reduction in removal efficiency, which aligns with the results observed in the present study (ciprofloxacin with a pKH of 16 and sulfamethoxazole with a pKH of 12) [25].

**Figure 4:**
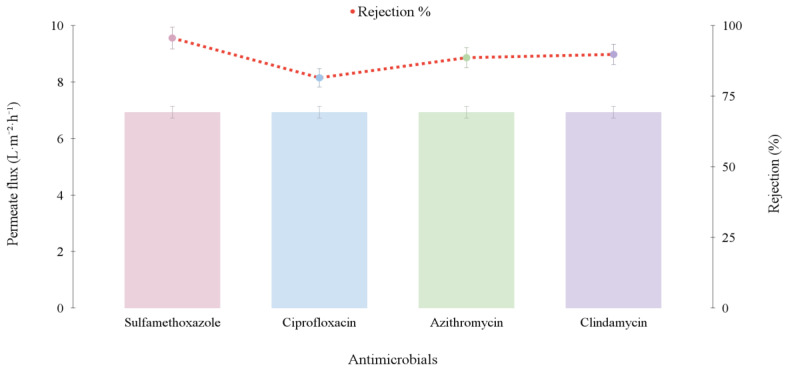
Permeate flux and rejection rate of each compound in a mixture. Test conditions: micropollutant concentration equal to 500 μg·L^−1^, temperature of 75 °C, vacuum pressure of −640 mmHg, and feed flow rate of 24 L·h^−1^.

In the test conducted with a feed solution containing all four compounds simultaneously, the permeate flux was 6.93 L·m^−2^·h^−1^, which showed no statistically significant difference (*p* > 0.05) compared to the fluxes obtained with the individual feed solutions. Additionally, Figure 4 presents the rejection results for each component in the solution.

The results suggest that complex interactions interfered with the rejection process, as the antimicrobials exhibited lower rejection rates when present in the mixed feed solution compared to when used in individual solutions. In the individual solutions, the rejection efficiency of the DMV system was close to 99%. However, in the mixed solutions containing all four antimicrobials, sulfamethoxazole showed the highest retention (95%), followed by clindamycin (88%), azithromycin (87%), and ciprofloxacin, which had the lowest rejection (80%). The results suggest that the complex interactions between the compounds contributed to the observed differences in rejection rates, with 95% rejection of sulfamethoxazole and 88% rejection of clindamycin - both slightly lower than the rejection rates observed for the antimicrobials when tested separately.

The role that the membrane played in each separation process was valid. The hydrophobic porous PTFE membrane in VMD acted only as support for the vapor–liquid interface and did not contribute to the separation performance. Contrarily, hydrophilic membranes facilitated the relative solubility and diffusivity of water across the membrane matrix [26]. Although PTFE (polytetrafluoroethylene) is a chemically inert material and does not possess charged functional groups, it can interact physically with charged molecules such as ciprofloxacin. Suppose ciprofloxacin is charged (for example, with a negative charge due to the ionization of the carboxyl group), it may be attracted to the membrane surface by electrostatic forces (opposite charge interactions). Thus, if ciprofloxacin is in a solution where it carries charges (such as -COO^−^), these charge interactions with the PTFE membrane surface may occur, though to a limited extent [27]. In addition, the hydrophobic interactions between the membrane and the targeted compounds and the varied porosity of the membranes can manage size exclusion, contributing to the enhanced removal of the targeted pharmaceutical molecules.

### 3.2. Plant Ecotoxicity Test

**Figure 5:**
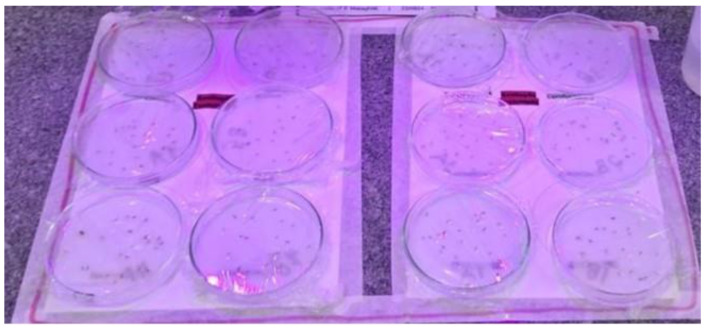
Image of Petri dishes containing *Lactuca Sativa* L. seeds. Image: 12 Petri dishes containing 20 *Lactuca Sativa L*. seeds in each under pink light and added to the solution to be toxicologically analyzed.

Figure 5 shows an image of the 8 Petri dishes containing 20 *Lactuca Sativa* L. seeds in each on qualitative filter paper substrate (Qualy: 80 g·m^2^, ⌀ of 90 mm) under pink light (LED Grow of 60 LEDs [pink: 19 blue LEDs, 41 red LEDs]), for 120 h of exposure, used to identify the behavior of the roots when exposed to the different antimicrobials at a concentration of 500 μg·L^−1^.

**Figure 6:**
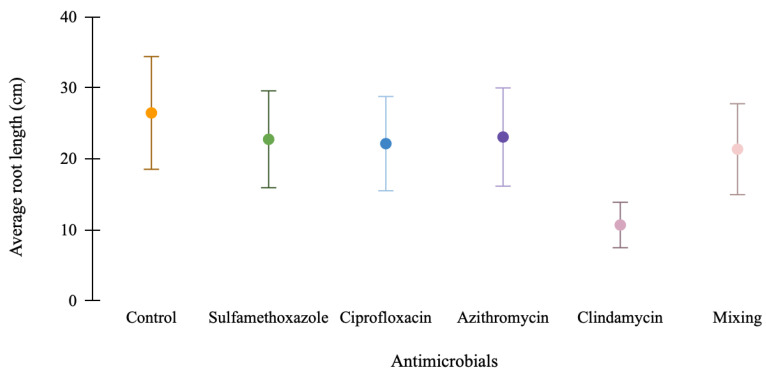
Average radicle length of *Lactuca sativa* L. under 120 h toxicity test.

**Figure 7:**
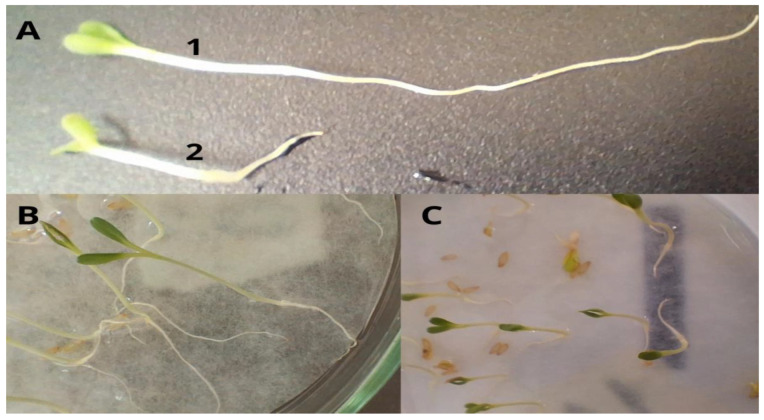
Comparative root image of *Lactuca sativa* L. at 120 h. Image: Rootlets at 120 h. (**A**): 1 control (average size 26.52 cm); 2 clindamycin (average size 10.68 cm). (**B**): control. (**C**): clindamycin.

The results regarding the average root length are shown in Figure 6. It can be observed that lettuce seeds exposed to antimicrobial agents did not exhibit a statistically significant difference (*p* > 0.05) in root length compared to those exposed only to water. The exception was the seeds exposed to clindamycin. Distilled water (control) presented the highest root length values, with an average of 26 cm. When the seeds were exposed to the clindamycin solution, a reduction in root growth was observed compared to the control, with root lengths nearing 10 cm. Thus, clindamycin was the antimicrobial that had the greatest impact on lettuce root development, a finding further supported by the photographs in Figure 7 and the toxicity index presented in Table 3 below.

Figure 7 shows the difference between a seed grown in distilled water and one grown in clindamycin solution for 120 h. In addition to rootlet length, other factors were evident in the test. It was observed that the natural shape of the rootlets followed a typical root pattern. However, the rootlets of plants exposed to clindamycin (classified as “high” toxicity) exhibited an altered pattern, likely due to underdevelopment. These rootlets appeared more linear, with no lateral roots emerging. This difference in size and shape is illustrated in Figure 7, which provides a comparison of the root structures.

Table 3 presents the analysis of germination and growth of L. sativa seeds, with the indices IG%, IGN%, and IER%. It can be observed that all antimicrobials exhibited some degree of toxicity, although considered low, with clindamycin showing the highest level of toxicity.

In this analysis, the *IER* values classified the antimicrobials into low toxicity levels (0 to −0.25) for sulfamethoxazole, ciprofloxacin, and azithromycin and high toxicity levels (−0.5 to −0.75) for clindamycin. The *IER* values classified the antimicrobials into low toxicity levels (0 to −0.25), indicating that these compounds had a minimal impact on seed germination and early root development. This classification suggests that sulfamethoxazole, ciprofloxacin, and azithromycin may exert limited inhibitory effects on the initial stages of plant growth, allowing the seeds to germinate and establish rootlets, albeit with a potential reduction in overall root development. The low toxicity levels imply that these antimicrobials could be less harmful to the plant system compared to other substances, potentially allowing for growth under controlled conditions. However, further investigations would be necessary to confirm their long-term effects on plant development and their environmental implications.

The *IER* values also enabled the classification of the antimicrobials based on *IG* or *IGN* levels. These indices assess germination, rather than root growth. Notably, even when exposed to highly toxic compounds, seeds may still germinate, indicating that the initial process of seed sprouting can occur despite inhibitory effects. However, this does not necessarily guarantee continued development, as the lack of proper root growth or other developmental processes can hinder the overall health and viability of the plants.

Therefore, by comparing the sizes and using the equations proposed by De Andrade et al. [19], the impact of antimicrobials on root and plant development is quite evident, particularly with clindamycin, which had a significant effect in inhibiting root growth. As a result, the plants exhibited limited root development, with the few roots that did grow taking on a more linear shape and lacking lateral structures branching from the main roots.

### 3.3. Human Risk Analysis

The estimated daily intake (*EDI*) of water for the Brazilian population is an average of 35 mL per kg per day (average of 2.2 kg·day^−1^ per adult person) [20]. Using this data, the estimated average daily dose (*ADD*) of emerging CEC contaminants was calculated. The values of the antimicrobial concentrations in the water of Lagoa da Conceição used for the risk calculations were obtained from Da Silva et al. [5] (Annex 1). These data were adapted and are listed in the complementary material.

The results based on the concentrations of the antimicrobials presented (Annex 1), the risk, and the hazard index (*HI*) for the compounds sulfamethoxazole, ciprofloxacin, azithromycin, and clindamycin are shown in Table 4 below.

The compounds ciprofloxacin and clindamycin present a risk that can vary from moderate to high, depending on the age group. Age is a crucial factor, as it is related to exposure time; the older the person, the longer the daily exposure time due to direct consumption. Additionally, the type of exposure is another important aspect to consider. Exposure can occur through intentional or unintentional ingestion, or even through superficial contact, with each type potentially leading to varying levels of absorption and effect. The route and frequency of exposure, combined with an individual’s age and mass, collectively influence the potential health risks associated with these compounds [28]. Although the results do not suggest an immediate risk to human health, the presence of emerging contaminants (ECCs) should be considered a public health concern. According to Burmaster and Anderson (1994), the risk must be on a logarithmic scale from 10^−6^ to 10^−4^ to be considered a low and moderate risk, respectively, above this value the risk would be high or very high [29]. Given the growing recognition of ECCs, it is crucial to develop comprehensive strategies that assess exposure to multiple contaminants simultaneously and evaluate the effects of their potential interactions. Ignoring these combined effects may lead to an underestimation of the true risks, making it essential to adopt a more integrated approach to environmental health assessment [30].

Considering the risk analysis outlined above, the study and implementation of preventive and corrective strategies to mitigate water contamination have become urgent. In terms of corrective actions, the use of DMV has shown promising potential for effectively removing the CECs investigated in this study. The results suggest that DMV could be a viable solution for reducing these contaminants under the conditions examined, offering a potential method for improving water quality and safeguarding public health.

## 4. Conclusions

Vacuum membrane distillation (VMD) is a technique with the potential to remove emerging contaminants, such as antimicrobials, which can be harmful to health and the environment. The results obtained in this study indicated a 99.9% retention of salt in saline water and average rejections of 99.1% for sulfamethoxazole, 98% for ciprofloxacin, 99.9% for azithromycin, and 99% for clindamycin when analyzed individually, demonstrating the effectiveness of VMD. This rejection showed a decrease when the antimicrobials were permeated simultaneously. Sulfamethoxazole showed the highest retention (95%), followed by clindamycin (88%), azithromycin (87%), and ciprofloxacin, which showed the lowest rejection (80%).

Ecotoxicity tests on lettuce (*Lactuca sativa* L.) showed low toxicity for most antimicrobials, except for clindamycin, which exhibited high toxicity. This underscores the importance of efficient processes to remove these harmful compounds from water. The human risk assessment in water revealed that the risk associated with ciprofloxacin (Hazard Index = 3.18 × 10^−4^) and clindamycin (Hazard Index = 3.53 × 10^−3^) ranges from moderate to high, respectively.

It is essential to adopt proactive measures to reduce and ensure the preservation and sustainability of aquatic ecosystems. Based on the results of this study, new approaches for the removal of contaminants of emerging concern (CECs), particularly antimicrobials, can be guided, highlighting the potential of VMD to remove organic contaminants and biocompounds from water, opening new possibilities for the treatment of contaminated water across the country.

## Figures and Tables

**Table 1:** Characteristics of the Fluoropore membrane (Merck Millipore).

	Characteristics
Polymer	PTFE
Pore size (µm)	0.22
Air flow (L·min^−1^·cm^−2^)	5
Water flow (mL·min·cm^−2^)	24
Porosity (%)	85
Thickness (µm)	150
Maximum Temperature (°C)	130
Wettability	Hydrophobic

**Table 2:** Saline rejection collected in the VDM tests at 65 and 75 °C and −640 mmHg.

	Saline Solution		Lagoa da Conceição Water	
	65 °C	75 °C	65 °C	75 °C
Rejection (%)	99.7 ± 0.3	99.8 ± 0.2	99.8 ± 0.2	99.9 ± 0.1
Permeate flux reduction (%)	6	22	11	36
Permeate flux (L·m^2^·h^−1^)	2.9 ± 0.2	7.9 ± 0.3	3.3 ± 0.2	8.2 ± 0.1
Conductivity (µ·Scm^−1^)	27,800		29,900	
Concentration (g·L^−1^)	27.14		30.01	

**Table 3:** Antimicrobial toxicity test of *Lactuca sativa* L. in 120 h.

Sample (Root)	Control (H_2_O)	Sulfamethoxazole	Ciprofloxacin	Azithromycin	Clindamycin	Mixing
Average size (cm)	26.52 ± 9.15	22.78 ± 5.60	22.24 ± 4.89	22.92 ± 6.09	10.68 ± 6.02	21.36 ± 4.95
Average number of ungerminated seeds	19	20	18	18	17.50	17.50
% Germination	95	95	90	90	87.50	87.50
*GI*%	-	90.40	79.42	81.88	37.10	74.16
*NGI*	-	0	−0.051	0	−0.028	0
*RSG*	-	105.26	94.74	94.74	92.11	92.11
*RGR*	-	85.87	83.84	86.43	40.28	80.52
*NGR*	-	−0.14	−0.16	−0.14	−0.60	−0.19
Toxicity	Control ◎	Low ◉	Low ◉	Low ◉	High ◉	Low ◉

Toxicity: *GI*: germination index. *NGI:* normalized residual germination percentage index. *RSG*: relative germination. *RGR*: relative radicle growth. *NGR*: normalized residual radical elongation percentage index. According to De Andrade et al. [16], *NGI* and *NGR* indicate the level of toxicity: 0 to −0.25: low toxicity; −0.25 to −0.5: moderate toxicity; −0.5 to −0.75: high toxicity; −0.75 to −1.0: very high toxicity; greater than 0: hormesis (beneficial dose for the test organism).

**Table 4:** Risk analysis and human hazard index of antimicrobials in concentrations close to that of Lagoa da Conceição water for 35 year old adults with an average weight of 70 kg.

	Sulfametoxazol	Ciprofloxacino	Azitromicina	Clindamicina
*ADD* (ng·kg^−1^/bw·day^−1^)	ND	226.65 ± 45.32	*ND*	211.5 ± 42.3
*HI* (ng·kg^−1^/bw·day^−1^)	ND	3.18 × 10^−4^	*ND*	3.53 × 10^−3^
Risk level		++		+++

*ADD*: estimated average daily dose. *HI*: hazard index. *ND* = not detected. Risk level: (+) negligible; (++) moderate; (+++) high.

## Data Availability

The data that support the findings of this study are available from the corresponding author upon reasonable request.

## Supplementary Materials

The following supporting information can be downloaded at: https://www.mdpi.com/article/10.3390/membranes15050127/s1, Table S1: CECs quantitative evaluation by HPLC-MS/MS of water samples collected in the Lagoa da Conceição region.
