# Supplementary material for: Addressing Contaminants of Emerging Concern in Aquaculture: A Vacuum Membrane Distillation Approach
Source: Membranes (Basel). 2025 Apr 24;15(5):127. doi: 10.3390/membranes15050127 (PMC12113035; doi:10.3390/membranes15050127)
Supplement: Supplementary file 1 [file membranes-15-00127-s001.zip › membranes-3522442-supplementary.pdf]

Table S1. CECs quantitative evaluation by HPLC-MS/MS of water, sediment, and fish samples collected in the Lagoa da Conceição region **[5]**.

Month/year collection	AMF	CAF	CIP	CLI	DCF	ERI	LIN	SER	SUT	TMP	PXC	MXC
WN 23/02	ND	ND	**ND**	**ND**	ND	ND	ND	ND	**ND**	ND	ND	ND
SN 23/02	ND	ND	**ND**	**ND**	ND	ND	ND	ND	**ND**	ND	ND	ND
WLE 23/04	ND	< LOQ	**ND**	**< LOQ**	ND	ND	ND	ND	**< LOQ**	< LOQ	ND	ND
12/22	ND	4.23 – 15.025	**6.047**	**6.41 – 7.05**	1.358 – 2.203	ND	ND	< LOQ	**ND**	ND	ND	ND
SLE 02/23	ND	7.799	**ND**	**ND**	ND	ND	ND	ND	**ND**	ND	ND	ND
04/23	ND	2.994 – 5.397	**ND**	**ND**	1.698	ND	ND	ND	**ND**	ND	ND	ND
WLC 1 23/04	ND	< LOQ	**ND**	**ND**	ND	ND	ND	ND	**ND**	ND	ND	ND
12/22	ND	2.612 – 23.696	**6.777**	**ND**	10.725	ND	ND	ND	**ND**	ND	ND	ND
SLC 1 02/23	ND	9.198 – 44.17	**6.953 – 7.543**	**ND**	ND	ND	ND	ND	**ND**	ND	ND	ND
04/23	ND	3.009 – 7.595	**ND**	**ND**	4.785	ND	ND	ND	**ND**	ND	ND	ND
WLC 2 23/04	< LOQ	< LOQ	**ND**	**< LOQ**	< LOQ	ND	ND	ND	**ND**	ND	ND	ND
12/22	ND	10.019 – 24.681	**3.336 – 6.104**	**ND**	1.995 – 4.181	ND	ND	ND	**ND**	ND	ND	ND
SLC 2 02/23	ND	26.716 – 31.646	**6.798**	**ND**	1.144	ND	ND	ND	**ND**	ND	ND	ND
04/23	3.016	6.047 – 9.55	**6.175**	**ND**	1.736	ND	ND	< LOQ	**ND**	ND	ND	ND
WLC 3 02/23	< LOQ	< LOQ	**ND**	**ND**	< LOQ	ND	ND	ND	**< LOQ**	< LOQ	ND	ND
04/23	ND	< LOQ	**ND**	**ND**	ND	ND	ND	ND	**ND**	ND	ND	ND
12/22	ND	10.413 – 14.909	**ND**	**1.695 – 1.771**	1.247	ND	ND	ND	**ND**	ND	ND	ND
SLC 3 02/23	ND	14.562 – 22.258	**6.763**	**ND**	ND	ND	ND	ND	**ND**	ND	ND	ND
04/23	1.682	33.518	**ND**	**ND**	23.772	ND	ND	ND	**ND**	ND	ND	ND
WLC 4 23/04	ND	< LOQ	**ND**	**ND**	ND	ND	ND	ND	**ND**	ND	ND	ND

AMF: acetaminophen; CAF: caffeine; CIP: ciprofloxacin; CLI: clindamycin; DCF: diclofenac; ERI: erythromycin; LIN: lincomycin; SER: sertraline; SUT: sulfamethoxazole; TMP: trimethoprim; PXC: piroxicam; MXC: meloxicam; <LOQ: less than the limit of quantification; ND: not detected. W: water samples; S: sediment samples; N: spring; LE: seepage lagoon of the wastewater treatment plant; LC1: corner of the lagoon; LC2: Center; LC3: Avenida das Rendeiras; LC4: Barra da Lagoa; LC5: Foz do Rio Vermelho; LC6: Morro dos Macacos; LC7: Costa da Lagoa. Antimicrobials highlighted in the blue lines were the analytes used in this study. Source: Adapted from Da Silva *et al*. **[5]**. Green columns were the data used for human risks for calculations.
